# New Technologies for Stroke Rehabilitation

**DOI:** 10.1155/2013/815814

**Published:** 2013-01-20

**Authors:** Marco Iosa, Stefan Hesse, Antonio Oliviero, Stefano Paolucci

**Affiliations:** ^1^Clinical Laboratory of Experimental Neurorehabilitation, Fondazione Santa Lucia, IRCCS, Via Ardeatina 306, 00179 Rome, Italy; ^2^Department of Neurological Rehabilitation, Medical Park Berlin, Charité-Universitätsmedizin Berlin, 13507 Berlin, Germany; ^3^Functional Exploration and Neuromodulation of Nervous System Investigation Group (FENNSI), Hospital Nacional de Paraplejicos, Finca La Peraleda s/n, 45071 Toledo, Spain; ^4^Operative Unit F, Fondazione Santa Lucia, IRCCS, Via Ardeatina 306, 00179 Rome, Italy

“Real progress happens only when advantages of a new technology become available to everybody” said Henry Ford; we would add “or to the most disadvantaged people.” 

Stroke is the leading cause of disability in all industrialized countries. Common rehabilitation usually allows about 50% of patients with stroke to recover walking, leaving the others not independent in walking and other activities of daily living [1]. For these reasons, an increasing number of researches are pursuing the use of new technologies to improve the efficacy of rehabilitation. For example, over the last decade, many devices for robotic-assisted training have been developed to allow patients to perform early, intensive, and task-oriented exercises [2, 3], and wearable devices for an objective assessment of human movements have been developed for substituting in clinical settings the expensive and difficulty to use stereophotogrammetric systems of research gait laboratories [4]. Moreover, invasive and noninvasive techniques allowing the manipulation of brain excitability and plasticity appeared in the last decades. If their promises will be confirmed in the next future, these techniques associated to rehabilitation may boost the recovery of stroke patients. 

Differently from other fields of engineering, studies about the effectiveness of these technologies often occur after their development and their insertion in the rehabilitation settings. A number of studies showed the efficacy of these new technological approaches, whereas some others did not show any improvement in respect of conventional therapies. This uncertainty about efficacy, together with high purchase cost for some of these devices, some difficulties in their use by untrained staff, the absence of clear guidelines about better dosage and parameter values to select, and a somewhat diffuse scepticism by some members of the rehabilitation teams may limit the transfer of these new technologies from research laboratories to clinical settings, where patients are waiting to benefit from them. 

This special issue aimed to provide an overview on the use of new technologies for the rehabilitation of people with stroke. It contains two reviews and seven original researches. There is a generic review of M. Iosa and colleagues about seven promising technologies for stroke rehabilitation: robots, brain computer interfaces, noninvasive brain stimulators, neuroprostheses, virtual reality, wearable devices, and tablet-pcs. A more specific review is that of E. Q. van Delden and colleagues about twenty different devices for bilateral upper limb training. Upper limb rehabilitation was also the object of the study of G. Thielman and P. Bonsall, reporting the combined use of different technologies such as a unilateral robotic device used for interacting with a virtual task on a monitor, together with an auditory biofeedback for improving the trunk control. Also the study of P. Sale et al. was focused on the use of robotic rehabilitation of upper limb, in their case using a unilateral device designed for hand rehabilitation. Positive outcomes were found in all these studies, suggesting that robotic devices may provide a useful extension of currently available forms of therapy, as also stated in the paper by E. Q. van Delden and colleagues. But this special issue was not only focused on robotic devices. A. R. Bowers and colleagues investigated the on-road detection performance by drivers with hemianopia using oblique peripheral prisms. The study of G. Morone and colleagues showed the potentiality of using a neuroprosthesis not only for correcting the foot drop in chronic phase, but also for improving the sensorimotor relearning during rehabilitation in subacute phase of stroke, facilitating the gait recovery. P. J. Manns and R. G. Haennel investigated the use of a wearable device for assessing walking capacity in people after stroke in terms of energy expenditure and step measurement, concluding that this device is not enough accurate as step counter. So not all technologies can be used also in stroke population, and not in a nonspecific manner. The study of A. Fusco and colleagues, in fact, analysed three different montages of the electrodes of transcranial direct current stimulation, suggesting different effects among anodal montage acting on manual dexterity, cathodal montage improving manual force, and bilateral montage, the less effective. Also, G. Thielman and P. Bonsall suggested that the degree of changes varied per protocol and may be due to the appropriateness of the technique chosen, as well as based on patients impairments.

These observations result in line with the proposal to change the research question from “is this specific technology effective?” into “how may I use this technology in an effective way?” as suggested by M. Iosa et al. [5] and “which patients is this technology effective for?” as suggested by G. Morone et al. [6]. Robotic devices, for example, were shown to be more effective for severely affected patients, whereas rehabilitative outcomes after robotic training resulted similar to those of conventional manual therapy for moderately affected patients [6, 7]. 

This special issue would contribute to provide clear results on the potential benefits of the use of new technologies for patients with stroke, avoiding overstatements of results, and, at the same time, reducing the scepticism of those who say “With machines they would perform miracles. What sort of miracles?” as asked by the Inquisitor in the drama of Bertolt Brecht *Life of Galileo*.

*Marco Iosa* *Marco Iosa* *Stefan Hesse* *Stefan Hesse* *Antonio Oliviero* *Antonio Oliviero* *Stefano Paolucci* *Stefano Paolucci*
